# Long-Term Outcomes in Patients with Stroke after in-Hospital Treatment—Study Protocol of the Prospective Stroke Cohort Augsburg (SCHANA Study)

**DOI:** 10.3390/medicina56060280

**Published:** 2020-06-06

**Authors:** Michael Ertl, Christa Meisinger, Jakob Linseisen, Sebastian-Edgar Baumeister, Philipp Zickler, Markus Naumann

**Affiliations:** 1 Department of Neurology and Clinical Neurophysiology, University Hospital Augsburg, Stenglinstr. 2, 86156 Augsburg, Germany; philipp.zickler@uk-augsburg.de (P.Z.); markus.naumann@uk-augsburg.de (M.N.); 2 Independent Research Group Clinical Epidemiology, Helmholtz Zentrum München, Ingolstädter Landstr. 1, 85764 Neuherberg, Germany; c.meisinger@unika-t.de (C.M.); J.Linseisen@unika-t.de (J.L.); s.baumeister@unika-t.de (S.-E.B.); 3 Chair of Epidemiology, Ludwig-Maximilians-Universität München, UNIKA-T Augsburg, Neusässer Str. 47, 86156 Augsburg, Germany

**Keywords:** stroke, health care, mortality, complications, recurrence, patient cohort

## Abstract

Introduction: In Germany, stroke is the third leading cause of death, with more than 60,000 fatalities out of approximately 260,000 cases (first-ever and recurrent strokes) each year. So far, there are only a few long-term studies investigating determinants of the natural course of the disease, especially in the era of mechanical thrombectomy. Materials and Methods: The prospective single-center stroke cohort Augsburg (SCHANA) study will include about 1000 patients treated for stroke in the University Hospital of Augsburg. Patients aged 18 years or older with a confirmed diagnosis of ischemic or hemorrhagic stroke are included in the study. Information on demographic characteristics, onset of symptoms, etiologic factors, comorbidities, quality of life, invasive and non-invasive treatment, complications, and laboratory parameters are collected during a personal interview conducted during the patients’ hospital stay and via a medical chart review. About 30 mL of blood is collected from each patient, and after processing and aliquoting, all blood specimens are frozen at −80° C. The study participants will be followed-up via postal questionnaires at three and 12 months after discharge from the hospital. Furthermore, mortality follow-ups will be conducted. Cox-regression analysis will be used to estimate relative risks. Conclusion: The SCHANA study will generate comprehensive data on the long-term course of the disease. In addition to the main outcomes, recurrent events and survival, patient-oriented outcomes such as health-related quality of life and depression are the focus of the study.

## 1. Introduction

According to the World Health Organization (WHO), approximately 15 million people suffer a stroke every year. A high proportion of the cases are fatal, and one-third of stroke patients are affected by a serious, permanent disability [1]. In Germany, stroke is the third-leading cause of death, with more than 60,000 fatalities out of approximately 260,000 cases (first-ever and recurrent strokes) each year [2]. Nearly three-quarters of all strokes occur in people over the age of 65, with the chances of complete recovery or good functional outcome diminishing with increasing age [3]. A high number of stroke survivors suffer post-stroke depression, which is characterized by lethargy, irritability, sleep disturbances, lowered self-esteem, and withdrawal [4]. Up to 10% of people develop seizures following a stroke; the severity of the stroke increases the likelihood of a seizure [5]. The health-related quality of life in stroke survivors shows large variability but is lower than that of the general population [6].

To improve the clinical outcome and recovery of acute ischemic stroke patients, the primary therapeutic goal is the timely restoration of blood flow to salvageable ischemic brain tissue that is not already infarcted [7]. Reperfusion therapy using thrombolysis and endovascular interventions such as mechanical thrombectomy (MT) are the only approved treatments for acute ischemic stroke. If possible, both methods are applied in an overlapping manner (“bridging” therapy), starting with systemic thrombolysis and followed by MT. Still, there are no randomized controlled trials that support additional benefits of tissue plasminogen activator (tPA) application with MT. The current study can help to examine this in more detail in terms of long-term processes and may contribute to exploring bridging therapy in addition to advanced imaging-based approaches to select appropriate patients.

Both thrombolysis and mechanical thrombectomy result in an overall benefit regarding living without disability, but they do not improve survival [8,9]. A portion of patients develop complications after thrombolysis or thrombectomy [10,11], and the neurological recovery is dependent on the patient’s baseline functional status, as well as the status of the brain tissue, as assessed by brain imaging. Thus far, it is not entirely clear which patients are more likely to suffer a complication and which are not. Studies conducted to answer the question of long-term outcome depending on bridging therapy or MT alone were either relatively small, and retrospective [12], or the follow-up period was relatively short (max. of 3 months) with the outcome measures focusing on the modified Rankin Scale alone [13]. The stroke cohort Augsburg (SCHANA) study will help to identify patient groups that are more likely to be affected by post-therapeutic complications.

The clinical distinction between ischemic (IS) (thrombotic or embolic) and hemorrhagic strokes (HS) is of great importance because the management of the various types is very different [3]. Patients with HS require supportive care and have to be monitored for blood pressure control, changes in the level of consciousness, and their blood sugar, and oxygenation must be kept at optimum levels. The prognosis of HS, in general, is worse than for patients with ischemic strokes, but still relatively little is known about the reasons for differences in the acute phase versus long-term outcomes, especially concerning the quality of life in both patient groups in correlation with their functional outcomes. Big registry data are available with the focus on risk factors and outcomes comparing IS and HS, revealing many similarities (age, sex, and hypertension) but also differences in, e.g., smoking and alcohol consumption favoring HS [14]. This highlights the importance of analyzing the risk factors of both IS and HS. However, even large registries focus only on “functional” outcome variables and mortality and omit health-related quality of life, fatigue or depression, and healthcare utilization. Furthermore, studies are increasingly focusing on the identification of biomarkers associated with risk factors and pathogenesis of stroke to make the prevention, diagnosis, and management of stroke more effective [15,16].

The SCHANA study will provide insight into the risk factors, diagnostic procedures, treatment options, and the long-term disease course of a stroke. Furthermore, new knowledge regarding the development, treatment, and prognosis of the disease will be generated by using the information on genetics and –omics data gained from blood samples. In addition, subgroup differences regarding treatment effects and patient outcomes such as long-term survival and recurrent events will be assessed. Patient-oriented outcomes such as quality of life, the burden of depressive disorders, and the extent of disability in patients with stroke will be investigated. Patient subgroups who would benefit from certain (novel) therapies will be identified, and knowledge about the safety and harm associated with the use of drugs will be generated. Specifically, the study will address the following aims:

### 1.1. Primary Outcome

To estimate the impact of thrombolysis, mechanical thrombectomy and drug treatment, comorbidity, treatment-related complications on stroke-related long-term survival, and to examine the frequency and time point of recurrent events; to examine interaction effects between thrombolysis, mechanical thrombectomy and drug treatment on the outcome.

### 1.2. Secondary Outcomes

1. To identify factors which are associated with the natural course of the disease.
2. To identify subgroups of stroke patients (by clinical, physiologic, or psychologic features) and to assess optimal in-hospital and outpatient treatment for these subgroups.
3. To explore the requirements of bridging therapy (thrombolysis and mechanical thrombectomy).
4. To evaluate the long-term health-related quality of life and the presence of emotional problems, anxiety and/or depression in stroke patients.
5. To measure and monitor the safety and harm associated with the use of thrombolysis, mechanical thrombectomy, and drug treatments in hospital and after discharge.
6. To investigate the role of blood biomarkers (e.g., inflammatory parameters, adipokines) and of “omics” information (genome, metabolome, and proteome data) in disease development, progression, and outcomes.
7. To determine the health care utilization of patients with stroke, and to determine differences in health care utilization according to age, gender, socio-economic status, and place of residence (urban vs. rural).

## 2. Methods and Analysis

The study includes all consecutive adult patients (18 years and older) with incident as well as recurrent ischemic and hemorrhagic stroke who are admitted to the University Hospital of Augsburg. After finishing the pilot study (July/August 2018), the main study began on 1 September, 2018, and is expected to end on 31 December, 2020 (recruitment phase and follow-up).

Participation in the study is voluntary, and a written, informed consent form has to be signed by patients who are willing to take part in the study. If a patient suffers from dementia or another disease with major restrictions, written informed consent is obtained by the responsible legal caregivers, and in this case, a proxy interview is conducted. An extra study information and consent form is provided for the legal caregivers. Patients with language difficulties are asked whether a relative is available for translating the consent form and answering the questions. If this is not possible, patients who are not able to understand the study information and consent form due to language difficulties, are excluded from the study. No further exclusion criteria are set. Patients with post-stroke cognitive impairment (defined by a mini-mental state examination score lower than 27) [17] are included in the study, but will be excluded from certain data analyses, which are exclusively based on questionnaire data provided by the patient.

This is also the case for the follow-up questionnaires. For patients with major restrictions, the responsible legal caregiver will receive the follow-up questionnaires to help the patient with filling in the questions or to answer the questions as a proxy.

At the University Hospital of Augsburg, a high number of stroke patients are treated-yearly about 1500 cases. Based on the experience in previous, comparable studies, it is expected that about 70 percent of these patients will participate. Data collection procedures are performed in accordance with the Declaration of Helsinki.

### 2.1. Data Collection at Baseline Hospital Stay

#### 2.1.1. Patient Interview and Chart Review

Trained study nurses prospectively record all stroke cases. Once the patients are on the general ward, they are informed about the study by a study nurse, who also delivers the study documents. Patients receive comprehensive and understandable information on the processes and consequences of participation in the study.

After the patients have signed the consent form, they are interviewed by a study nurse. In the interview, demographic information, symptoms upon presentation, diagnosis, predisposing and life-style factors, and co-morbidities (e.g., carcinoma, cardiac comorbidity, diabetes mellitus) are assessed. Clinical data on comorbidities, risk factors, medication prescribed prior to the hospital stay and at hospital discharge, diagnostic procedures, clinical characteristics, disabilities, laboratory parameters, and treatment regimens during the hospital stay are assessed by chart review (Table 1).

#### 2.1.2. Collection of Biomaterial

During the hospital stay, about 30 mL of blood is collected from each patient. The blood samples are processed and aliquoted into sample tubes according to a standardized procedure. The aliquots are subsequently frozen at −80 °C and stored. It is planned to measure pro- and anti-inflammatory cytokines (e.g., interleukin (IL)-6, tumor necrosis factor (TNF)-alpha, IL-4, IL-10), adipokines (e.g., adiponectin, chemerin), neuroimmunokines (e.g., brain-derived growth factor, neurotrophins) and metabolomics in blood samples

### 2.2. Follow-Up of the Stroke Patients

#### Morbidity Follow-Up and Update of Treatment, Lifestyle and Health Factors

**Figure 1:**
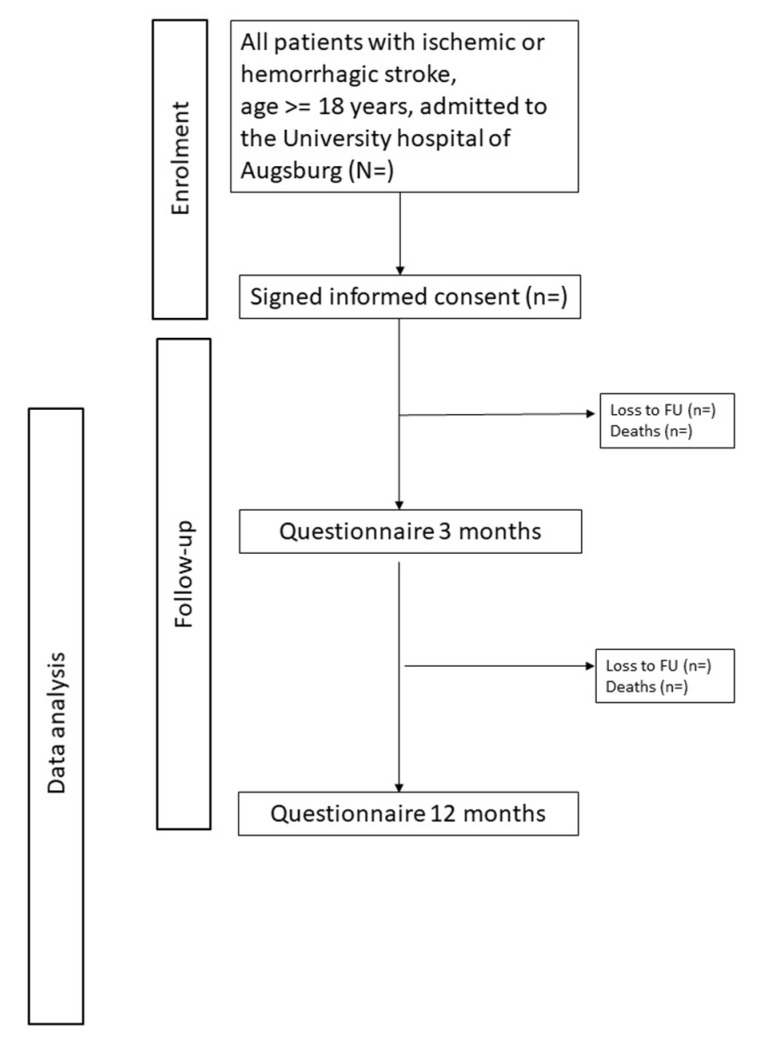
Consort chart of patient enrollment and follow up.

Three and 12 months after hospital discharge, all study participants will receive postal questionnaires or telephone interviews. The postal questionnaires will include questions on disease symptoms, complications, disabilities and comorbidities; behavioral and clinical risk factors (smoking, alcohol consumption, body weight, height); physical activity (German-PAQ-50+) [18]; health-related quality of life (Stroke Impact Scale) [19,20,21] and EQ-5D [22,23]; fatigue and headache (Rostock Headache Questionnaire) [24]; vertigo and depression (PHQ-9) [25,26]; recurrent events, bleeding outcomes, and healthcare utilization (readmissions, visits to physicians, clinics, and emergency departments); and data on current medication. An overview of the data collected at baseline and follow-up is given in Table 1, and a study flow-chart with enrollment and follow-up is depicted in Figure 1.

### 2.3. Mortality Follow-Up

Information on deaths occurring in the study sample during follow-up will be collected by conducting regular mortality follow-ups. The death certificates will be provided by the local health authorities, and the causes of death will be determined according to the International Classification of Diseases (ICD)-10 (WHO).

## 3. Statistical Analysis

Baseline characteristics will be presented as mean ± standard deviation (SD) or as a median and interquartile range for continuous variables; categorical variables will be given as percentages. Patient groups will be compared by Chi-squared test or Fisher’s exact test in case of categorical variables and by *t*-test for in case of continuous variables.

Differences between three or more patient subgroups will be conducted through log-linear models and analysis of variance, and if there are differences, they will be identified using post-hoc-tests.

Cox-Regressions will be carried out for the identification of risk factors and determinants which are related to the binary study outcomes. Confounder-adjusted models will be calculated to estimate relative risks. If age and sex are effect modifiers will be examined. Linear regressions will be applied in case of continuous outcomes if the model assumptions are fulfilled.

An alpha level of 0.05 will be considered significant within the multivariable analyses. In case of multiple testing, the significance level alpha will be corrected. The statistical analysis will be carried out using SAS software, SAS Institute, Cary, NC, USA and R project version 3.5.3, Vienna, Austria.

## 4. Sample Size Estimation

At the University Hospital of Augsburg yearly, about 1500 patients with acute stroke are treated. Expecting a study participation of about 70% it can be assumed, that altogether about 900 to 1000 patients will be recruited within one year. Based on a previous meta-analysis [27], a cumulative risk of stroke recurrence of 11% within one year can be expected. With an estimated hazard ratio (HR) of 1.7 for the covariate of interest, a variance of 0.36 and a rho^2^ = 0.3, at least 997 patients have to be included to find significant differences with a statistical power of 80% (significance level 5%).

## 5. Ethics and Dissemination

The study protocol was approved by the Ethics Committee of the Ludwig-Maximilians-Universität München (date of approval: 29.08.2018, Reference number: 18-196). The study is performed according to the Declaration of Helsinki. Written informed consent is obtained from each study participant. Patients who do not consent or are unable to consent are not be included, except for those for which we obtain consent by the legal caregivers.

## 6. Discussion

In this manuscript, the protocol for a long-term prospective observational cohort study, including patients with acute stroke treated in a hospital, is presented. In this study, the factors associated with the long-term course of this patient group will be investigated. At baseline, data on comorbidities, lifestyle factors, invasive and non-invasive diagnostic and treatment procedures as well as complications during a hospital stay is collected by a personal interview and a chart review. Three and twelve months after hospital discharge, follow-up data will be gathered via postal questionnaires. Furthermore, cause-specific mortality will be assessed through regular mortality follow-up.

Strengths of the present study are the large sample size, the generation of a considerable amount of data, in particular outcome data, and the long-term aspect of the study. Furthermore, data on the occurrence of the acute event are gathered through a face-to-face interview with the patients. Finally, from the study participants, blood is collected and stored. However, the study also has limitations. It is possible that selection bias will occur because patients who are more ill may refuse participation in the study. The present study is designed as a single-center and not as a multicenter study. Finally, another limitation is expected to be a loss to follow-up and the occurrence of missing data.

Altogether, the SCHANA study will add to the current knowledge on the long-term course of stroke and its subtypes. Through this study, prognostic factors regarding different outcomes will be examined. Due to the availability of blood specimens, novel biomarkers which may be associated with the disease course can be identified. Therefore, the results of the study might help guide the various disciplines involved in treating patients with stroke.

## Figures and Tables

**Table 1:** Data collected at baseline and during the follow-up.

Patient-Related Characteristics	Baseline Examination	Follow-Up after 3 Months	Follow-Up after 12 Months
Age	X		
Sex	X		
Place of residence	X	X	X
Nationality	X		
Marital status	X		
Education	X		
Occupation	X	X	X
Smoking	X	X	X
Alcohol consumption	X	x	x
Physical activity			
Female health (pregnancy, childbirth)	X	x	x
Pre-existing diseases	X	X	X
Prior treatments	X		
Family history of stroke	X		
Start of stroke symptoms	X		
(Prior) medication	X	X	X
Vital signs at hospital admission	X		
Treatment-related variables	X		
Date and time starting treatment prior admission	X		
Diagnostic procedures	X		
TOAST classification	X		
In-hospital treatment	X		
In-hospital complications	X		
Medication	X		
Treatment (e.g., thrombolysis)	x		
Laboratory values	X		
General parameters			
Date and time of hospital admission	X		
Date of hospital discharge	X		
Treatment at ICU and/or stroke unit			
Outcome parameters			
Health related quality of life		X	X
Disabilities			
Mental health		X	X
Fatigue			
Health care utilization		X	X
Medication		X	
Recurrent event		X	X
Bleeding complications		X	X
Headache			
Vertigo			
Dietary intake	X	X	X

## Abbreviations

CT	computed tomography
CTA	computed tomography angiographyv
EQ-5D	EuroQol-5D
HS	hemorrhagic stroke
ICD-10	International Classification of Diseases version 10
mRS	modified rankin scale
MT	mechanical thrombectomy
NIHSS	National Institutes of Health Stroke Scale
PAQ	physical activity questionnaire
PHQ	patient health questionnaire
SCHANA	stroke cohort Augsburg
TOAST	trial of ORG 10172 in acute stroke treatment
tPA	tissue plasminogen activator
SAS	statistical analysis system
WHO	World Health Organization
